# Enhancing Hippocampal Subfield Visualization Through Deep Learning Reconstructed MRI Scans

**DOI:** 10.3390/diagnostics15121523

**Published:** 2025-06-16

**Authors:** Nikolaus Clodi, Benjamin Bender, Gretha Hecke, Karolin Hauptvogel, Georg Gohla, Till-Karsten Hauser, Patrick Ghibes, Klaus Hergan, Ulrike Ernemann, Arne Estler

**Affiliations:** 1 Department of Radiology, Paracelsus Medical University of Salzburg, 5020 Salzburg, Austria; 2 Department of Diagnostic and Interventional Neuroradiology, University Hospital Tübingen, 72076 Tübingen, Germanytill-karsten.hauser@med.uni-tuebingen.de (T.-K.H.); ulrike.ernemann@med.uni-tuebingen.de (U.E.); 3 Nürnberg Hospital, 90419 Nürnberg, Germany

**Keywords:** magnetic resonance tomography, deep learning, epilepsy, hippocampus, FreeSurfer

## Abstract

**Background/Objectives:** Assessing hippocampal pathology in epilepsy is challenging, and improving diagnostic accuracy can benefit from deep learning image reconstruction, standardized imaging protocols, and advanced post-processing methods. This study compares T2 TSE DRB (Deep Resolve Boost) sequences with standard T2 TSE sequences for hippocampal segmentation and volumetry using FreeSurfer, focusing on how DRB affects image acquisition time without compromising diagnostic accuracy. **Methods:** FreeSurfer (version 7.4.1) was used to segment hippocampal subregions in 36 subjects (mean age of 39 ± 14 years; 21 males, 15 females) using both T2 TSE DRB and T2 TSE sequences. The segmented volumes were compared with a two-tailed *t*-test, and pathological volume differences were assessed using z-values based on a 95% confidence interval (−2 < z < 2). **Results:** Overall hippocampal segment volumes were identical between sequences. However, significant volume differences were noted in the CA1-Body (*p* = 0.003), CA4-Body (*p* = 0.002), and whole hippocampal body (*p* = 0.012) in the right hippocampus. Despite these differences, the low effect sizes suggest DRB sequences are comparable to conventional sequences. Additionally, DRB reduced image acquisition time by 61%. Z-scores identified pathological volume changes between the left and right hippocampus in individual subjects. **Conclusions:** T2 TSE DRB sequences are non-inferior to conventional T2 TSE sequences for hippocampal segmentation. The DRB method improves efficiency while providing clinically reliable results, and the proposed 95% confidence interval can aid in more objective assessments of hippocampal pathology.

## 1. Introduction

Magnetic resonance imaging (MRI) is a widely applied imaging modality used in the detection of lesions within the hippocampus in patients suffering from temporal lobe epilepsy [1]. Notably, 77% of patients experiencing first-time seizures present with either not visible or difficult-to-detect epileptogenic lesions. Timely treatment may result in higher rates of epilepsy control and patient satisfaction. Therefore, rapid diagnosis is required [2]. However, small lesions are only detected with a sensitivity of 39% due to not optimized usage of MRI sequences, failure to obtain precise information about clinical findings, and the lack of reader expertise [3]. In order to decrease the probability of detection failures, the HARNESS-MRI protocol, which comprises 3D T1, 3D FLAIR, and a high-resolution 2D T2 sequence in coronal slices along the hippocampus, should be considered when screening for hippocampal lesions [1]. Unfortunately, high-resolution images may lead to a decreased signal-to-noise ratio (SNR) and additional motion artifacts due to increased scan times [4]. To address this issue, deep learning reconstruction techniques have been proposed. Studies have already demonstrated that increased SNR and CNR (contrast-to-noise ratio) and decreased blurring can be achieved using such algorithms [5,6,7]. Despite recent advances in imaging technology, the diagnostic confidence for identifying pathological lesions remains low, with a reported certainty often below 50% [1,8]. To facilitate reliable comparisons of hippocampal volumes between healthy and diseased individuals and to promote the objective interpretation of MRI scans, several post-processing techniques have been developed. These include volumetric segmentation of hippocampal structures, T2 relaxometry, and texture analysis. T2 relaxometry, for example, provides a quantitative assessment of hippocampal tissues by measuring T2 relaxation times, which reflect water content and underlying microstructural properties. This technique typically utilizes dual-echo sequences, with the subsequent generation of T2 maps through voxel-wise fitting of exponential decay functions. Prolonged T2 values were found to be associated with pathological changes, such as gliosis, neuronal loss, or increased interstitial fluid. In parallel, a texture analysis evaluates the spatial distribution of signal intensities within MRI images, quantifying tissue heterogeneity through statistical descriptors of local intensity variation. When, for example, applied to focal cortical dysplasia, this method generates voxel-based feature maps capturing cortical thickness, gray-white matter junction blurring, and relative signal intensity differences, subtle features that may elude conventional imaging. While volumetric analyses of the hippocampus have traditionally focused on total volume or larger anatomical segments, detailed comparisons at the level of individual subregions are not routinely performed [5,6,9,10,11,12,13]. Previous studies have demonstrated that hippocampal subfield volume segmentation using FreeSurfer (Laboratories for Computational Neuroimaging, Athinoula A. Martinos Center for Biomedical Imaging, Charlestown, MA, USA) can aid in seizure lateralization and enhance the detection rate of epileptogenic lesions in patients with mesial temporal lobe epilepsy (mTLE). These studies, however, only utilize standard T1-weighted turbo spin echo (T1 TSE) and T2-weighted turbo spin echo (T2 TSE) sequences as input for the FreeSurfer analysis [9,14]. This study investigates objectively whether accelerated T2 TSE DRB sequences can serve as a viable alternative to the conventional T2 TSE sequence in hippocampal subregion volumetry, aiming to reduce imaging time without compromising the diagnostic accuracy. Furthermore, we explore whether hippocampal subfield volumes in healthy controls can help identify relevant volume changes in individual epilepsy patients, potentially enhancing lesion detection and improving clinical outcomes.

## 2. Materials & Methods

### 2.1. Study Design

This retrospective, single-center study received approval from the local review board with informed consent waived (code 161/2023B01). The study adhered to the principles outlined in the Declaration of Helsinki guidelines and recommendations. Over a period of 10 months (June 2023–March 2024), 36 patients were included in the study. All patients met the following inclusion criteria: they were scanned using an MRT MAGNETOM Vida Fit 3T (Erlangen, Germany) with the following sequences: T1w 3D, T2w TSE, and T2w TSE DRB. If a hippocampal pathology was detected, further classification into subgroups was conducted.

### 2.2. Patient Characteristics

A total of 36 patients with a mean age of 39 ± 14 years (15 females and 21 males, ranging from 21 to 72 years) were included in the study. Among them, 20 were healthy controls (pathological cases were gathered retrospectively; healthy controls were enrolled prospectively), and 16 had epilepsy, with 5 of them exhibiting visible lesions in the hippocampus. Two of these individuals suffered from postictal edema, two suffered from focal cortical dysplasia, and one suffered from hippocampal sclerosis. The remaining 11 epilepsy patients had non-lesional epilepsy at the time of the scan. Further details can be drawn from Table 1.

### 2.3. MRI Acquisition Parameters

All scans were performed on a Siemens Healthineers MRT MAGNETOM Vida Fit 3T (Erlangen, Germany) with 20-channel Siemens HeadNeck_20_TCS coils. The hippocampal MRI sequence protocol was selected to be consistent with current recommendations for hippocampal imaging as proposed by the International League Against Epilepsy Neuroimaging Task Force [1,15]. All sequences recommended by the task force that were amenable to acceleration via the DRB algorithm were accelerated; in our study, DRB reconstruction was applicable in the T2 2D TSE sequence.

Primarily, a T1 3D MPRAGE sequence was used for whole-brain segmentation employing FreeSurfer. Secondarily, two T2 TSE 2D sequences were acquired, which were used for hippocampal segmentation (see Table 2). The main difference between these two T2 TSE sequences was the way the data acquisition and reconstruction were performed. One of them, the conventional 2D T2 TSE sequence, was reconstructed using GRAPPA and accelerated using two phase encoding steps (acquisition time of 3:51), while the DRB 2D T2 TSE was accelerated four times using GRAPPA (acquisition time of 2:37) and subsequently reconstructed using the newly available deep learning reconstruction algorithm (DRB—Deep Resolve Boost) proposed by Siemens Healthineers (Erlangen, Germany) [5,6,7]. We optimized our imaging parameters by systematically analyzing the accelerated sequence using spine and brain images. Our iterative testing indicated that employing an acceleration factor of 4 with the newly available DRB reconstruction algorithm produced the most reproducible results while preserving a high signal-to-noise ratio. The comparison used a standardized sequence using an acceleration factor of R = 2, a parameter validated with GRAPPA reconstruction that has been previously applied for brain imaging at the University of Tübingen.

Both T2-weighted hippocampal scans were coarsely aligned to the T1-weighted scan and used for segmentation of hippocampal subregions and combined labels utilizing the FreeSurfer pipeline.

**Figure 1:**
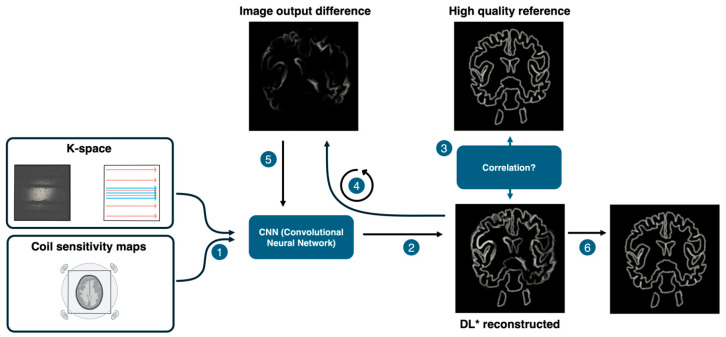
K-space data and coil sensitivity maps are input into a convolutional neural network (CNN). The CNN reconstructs an image, which is then compared to a high-quality reference. The resulting error (image output difference) is iteratively fed back into the network to refine the reconstruction. After training converges, a final image is produced with fixed model parameters [5,6,7]. * Deep Learning.

Deep Resolve Boost (DRB) is an FDA-approved deep learning-based image reconstruction method offered by Siemens Healthineers. It utilizes a convolutional neural network trained on a diverse set of MRI images to enhance image quality from undersampled raw data. A significant advantage of DRB is its compatibility with Siemens MRI systems, allowing immediate integration into clinical workflows (Figure 1) [5,6,7].

### 2.4. Image Evaluation Using FreeSurfer

**Figure 2:**
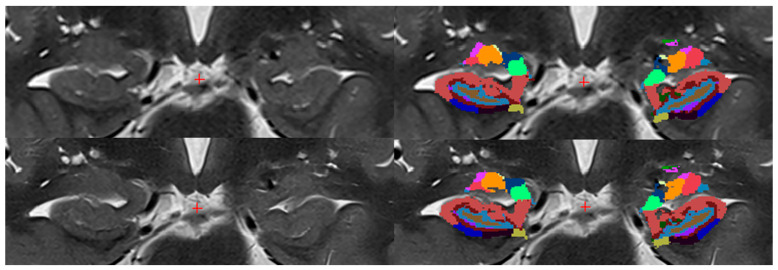
Hippocampal segmentation using FreeSurfer (healthy subject). The figure displays segmented hippocampal regions within a healthy individual using FreeSurfer. The left side shows images before segmentation, while the right side shows images after segmentation. The top row presents images obtained with the conventional T2 TSE sequence, while the bottom row presents images obtained with the T2 TSE DRB sequence. Hippocampal regions by color: 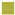 parasubiculum, 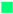 HATA, 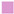 fimbria, 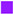 hippacampal_fissure, 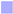 HP_tail, 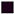 presubiculum-head, 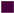 presubiculum-body, 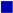 subiculum-head, 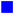 subiculum-body, 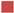 CA1-head, 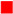 CA1-body, 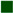 CA3-head, 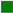 CA3-body, 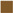 CA4-head, 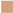 CA4-body, 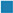 GC-ML-DG-head, 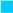 GC-ML-DG-body, 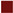 molecular_layer_HP-head, 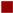 moleculcular_layer_HP-body.

**Figure 3:**
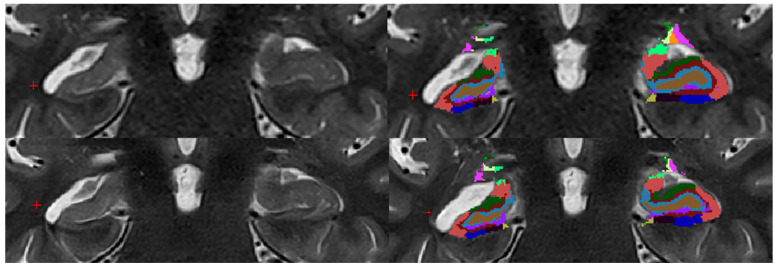
Hippocampal segmentation using FreeSurfer (HS). The figure displays segmented hippocampal regions in a patient with right-sided hippocampal sclerosis (HS), processed using FreeSurfer. The left side shows images before segmentation, while the right side shows images after segmentation. The top row presents images obtained using the conventional T2 TSE sequence, and the bottom row presents images obtained using the T2 TSE DRB sequence. Hippocampal regions by color: 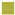 parasubiculum, 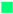 HATA, 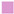 fimbria, 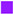 hippacampal_fissure, 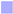 HP_tail, 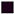 presubiculum-head, 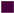 presubiculum-body, 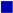 subiculum-head, 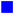 subiculum-body, 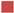 CA1-head, 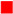 CA1-body, 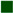 CA3-head, 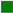 CA3-body, 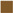 CA4-head, 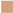 CA4-body, 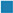 GC-ML-DG-head, 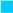 GC-ML-DG-body, 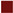 molecular_layer_HP-head, 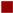 moleculcular_layer_HP-body.

Using FreeSurfer (version 7.4.1; Laboratories for Computational Neuroimaging, Athinoula A. Martinos Center for Biomedical Imaging, Charlestown, MA, USA), a segmentation of hippocampal subregions and combined labels was performed (Figure 2 and Figure 3) [16,17]. To ensure accuracy, three radiologists manually reviewed the automated segmentations for potential errors, thereby verifying consistency and reliability. Segmented volumes were compared between hippocampal regions delineated using conventional T2 TSE 2D and T2 TSE 2D DRB sequences, thus facilitating an objective evaluation. Both conventional T2 TSE and T2 TSE DRB images were co-registered with T1-weighted 3D MPRAGE images, which served as the anatomical reference and were employed for whole-brain segmentation (3D sequence), a requisite preliminary step prior to individual hippocampal region segmentation. Importantly, the segmentation of hippocampal subfields was exclusively based on T2-weighted imaging data. Additionally, given that the MPRAGE sequence is widely utilized in brain imaging for its superior delineation of white and gray matter and its well-established characteristics in brain region segmentation, it also served as a reference standard to verify the accuracy of the segmentation process.

**Figure 4:**
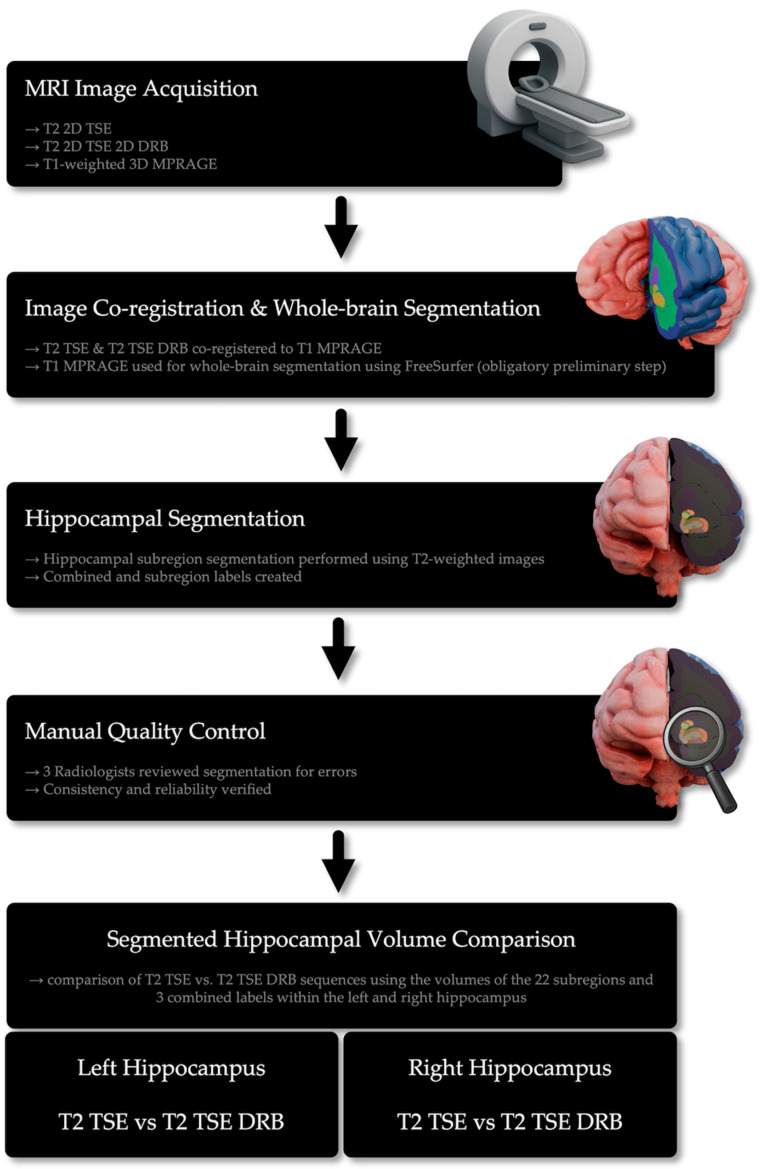
Overview of the workflow for hippocampal subregion segmentation and analysis using FreeSurfer. The process includes MRI acquisition, image co-registration, segmentation, manual quality control, and volume comparison between T2 TSE and T2 TSE DRB sequences using the volumes of hippocampal segments.

In the process, every hippocampal region was compared twice, using the left and right hippocampus in every subject. Due to organizational reasons, all 22 segmented hippocampal regions were classified as one of the following merged labels—HEAD, BODY, TAIL, and FISSURE—as suggested by FreeSurfer. In that context, the proposed merged labels included the following subregions, respectively: HEAD—Parasubiculum, Presubiculum, Subiculum-Head, CA1-Head, CA3-Head, CA4-Head, GC-ML-DG-Head, molecular_layer_HP-head, HATA; BODY—Presubiculum-body, Subiculum-body, CA1-Body, CA3-Body, CA4-Body, GC-ML-DG-Body, molecular_layer_HP-body, fimbria; TAIL—Hippocampal_tail; FISSURE—hippocampal-fissure. Furthermore, combined labels that cover the entire hippocampus were proposed and defined as follows: whole_hippocampal_body, whole_hippocampal_head, and whole_hippocampus [16]. Image segmentation was performed on a high-performance computing server at the University of Tübingen (80 CPU cores, 375.5 GB RAM) (see Figure 4).

### 2.5. Statistical Analysis

The manuscript was written using Microsoft^®^ Word for Mac, Version 16.97.2 (Build 25052611), and the statistical analysis was carried out using the commercially available software Microsoft^®^ Excel for Mac, Version 16.83 (Build 24031120) [18]. After a successful segmentation of the hippocampal subregions using the conventional 2D T2 TSE and the DRB 2D T2 TSE sequence, a paired comparison using a two-tailed *t*-test was performed to compare the obtained hippocampal volumes of all segments combined and of every segment individually [16]. Non-normally distributed parametric variables, as was the case when comparing the variance of regions between both sequences (assessed using the Shapiro–Wilk test), were analyzed using the Wilcoxon signed-rank test. Before the interpretation, *p*-values were corrected using the Holm–Bonferroni correction to account for multiple comparisons across 19 subregions and 3 combined labels within the left and right sides of the hippocampus. Compared to the standard Bonferroni method, the Holm–Bonferroni procedure controls the family-wise error rate while reducing the risk of Type II errors, making it more suitable for analyses involving a large number of related tests. The *p*-values exceeding 1 after correction were adjusted to 1. To assess the power of the results, a Cohen’s d test was applied to significant outcomes. Additionally, two board-certified neuroradiologists reviewed all of the MRI images in a blinded and randomized order. They were instructed to assess each image for blurring attributable to motion artifacts and to mark all images where such artifacts were observed. The resulting data were then compiled and are summarized in Table 3. In this study, we assume that the choice of MRI sequence does not significantly affect the measured hippocampal volumes using FreeSurfer. Accordingly, the null hypothesis (H_0_) states that there is no difference in hippocampal volumes, both in the right and left hippocampus, when measured using the standardized T2 2D TSE sequence compared to the T2 2D TSE DRB sequence. The alternative hypothesis (H_1_) posits that a significant difference does exist between the two sequences in terms of the measured volumes within the right and/or left hippocampus.

### 2.6. Setting a 95% CI for Hippocampal Pathology Detection

To establish an interval indicative of pathological volume differences between hippocampi, we first confirmed the absence of significant differences between the right and left hippocampal volumes in healthy individuals by conducting a paired *t*-test. We examine the potential lateralization of hippocampal volume within individuals. The null hypothesis (H_0_) assumes that there is no difference between the volumes of the left and right hippocampus in an individual. In contrast, the alternative hypothesis (H_1_) posits that a biologically and clinically meaningful difference exists between the left and right hippocampal volumes, indicating lateralization. Subsequently, we calculated the mean and standard deviation of the volume differences between the right and left hippocampus across individual hippocampal segments, utilizing data from 20 healthy controls in our cohort [19]. These values were then used to calculate a z-score using the following formula: $z=\frac{\left(right\;hippocampal\;region_{patient}-left\;hippompal\;region_{patient}\;\right)-Mean_{healthy}}{Standard\;deviation_{healthy}}$ for hippocampal volume differences in patients. The Mean_healthy_ and Standard deviation_healthy_ values can be taken from Table 4. A z-score outside the range of −2 < z < 2 indicates pathological volume differences between segments of the right and left hippocampus in an individual, corresponding to a 95% confidence interval (−2 < z < 2). Normally, when not assessing intra-individual volume discrepancies, one would need to control for global influences on hippocampal size, including age, sex, and brain volume [20]. However, as we compared intra-individual volume differences between the left and right hippocampus and not absolute volumes across individuals, our approach inherently controls for these factors. This eliminates the need for external adjustments and allows for the detection of pathological asymmetry based on the assumption of hippocampal symmetry in healthy controls, as confirmed by our paired *t*-test.

The literature remains inconclusive on whether a significant difference exists between the left and right hippocampus within an individual. This uncertainty arises due to varying hippocampal measurement methods, such as total volume, cross-sectional area, or specific subregions. Our study, focusing on total hippocampal volume as well as subregional volumes, aligns with others that highlight the importance of assessing the entire structure. These studies report no significant size difference between the left and right hippocampus. Others suggest a size difference most often linked to handedness; however, there is no general consensus on this matter [19,21].

## 3. Results

The examination of all regions combined within the hippocampus did not reveal any statistically significant differences between the T2 TSE sequence and the T2 TSE DRB sequence. Additionally, the comparison of variance between these sequences yielded non-significant results, suggesting comparable variability across both sequences. Notably, the T2 TSE DRB sequence achieved image acquisition 61% faster than the T2 TSE sequence.

However, when scrutinizing individual subregions and combined labels, significant disparities emerged in the CA1-Body, CA4-Body, and whole_hippocampal_body regions of the right hippocampus. Specifically, the *p*-values for CA1-Body, CA4-Body, and whole_hippocampal_body were observed to be *p* = 0.003, *p* = 0.0002, and *p* = 0.012, respectively, underscoring the statistical significance after the Holms–Bonferroni correction (see Table 5). Despite the observed significance, the associated statistical power was found to be notably low for all significant results: CA1-Body Cohen’s d = 0.0827; CA4-Body Cohen’s d = 0.1501; whole_hippocampal_body Cohen’s d = 0.0149.

Conversely, no concerning differences were noted when comparing the left and right hippocampus of healthy individuals, which was pertinent for the creation of an interval that should aid (using a 95% CI) in the detection of pathological volume discrepancies between the left and right hippocampus in an individual. The analysis, which was performed using T2 TSE and T2 TSE DRB sequences, yielded the following results: Significant volume disparities were solely detected in a few hippocampal regions, notably the Presubiculum-body (*p* = 0.0179; Cohen’s d = 0.8612) and Subiculum-body (*p* = 0.0037; Cohen’s d = 0.5498), when utilizing the T2 TSE sequence. Conversely, the examination of volume discrepancies via the T2 TSE DRB sequence identified a notable distinction only within the segment Presubiculum-body (*p* = 0.045; Cohen’s d = 0.8391). Noteworthy is the absence of significant differences in all other regions examined across both sequences when assessing volumetric variations between the left and right hippocampus in an individual (see Table 6).

Given that only a minority (3 out of 44) of the segments exhibited significant differences, it was inferred that there is no discernible global distinction between the left and right hippocampus in healthy individuals within our cohort.

To evaluate the effectiveness of pathological volume detection using z-values and utilizing a 95% CI, we calculated the z-values for each subregion and combined the labels for patients in our cohort using both the conventional T2 TSE sequence and the accelerated T2 TSE DRB sequence. The results are as follows:

In both sequences, the patient suffering from hippocampal sclerosis (HS) was successfully identified, with z-values below −2 across all combined labels, indicating significant atrophy of the right hippocampus (see Table 6). However, upon examining individual subregions, significant differences were detected in 15 out of 19 subregions with the T2 TSE DRB sequence, compared to only 13 out of 19 subregions with the conventional T2 TSE sequence. Similarly, in the patient with focal cortical dysplasia and concurrent edema within the left hippocampus, all combined labels were classified as pathological using z-values in both sequences. However, when evaluating individual subregions, the T2 TSE DRB sequence detected abnormalities in 8 out of 19 subregions, which was 2 more than those identified with the conventional T2 TSE sequence. In one patient suffering solely from right-sided hippocampal edema, both sequences identified the same number of affected subregions (4 out of 19) and combined labels (2 out of 3). For patients with epilepsy without previously detected hippocampal pathology, both sequences detected abnormalities in 1–2/19 segments analyzing the subregions in six patients, and no significant volume differences were observed in the combined labels for any of the patients. Notably, the z-values calculated using the conventional T2 TSE sequence identified one additional subregion as pathological in one patient, which was not detected by the T2 TSE DRB sequence. Furthermore, we assessed a patient with focal cortical dysplasia limited to the temporal lobe and part of the right hippocampus. In this case, no significant volume differences were detected between the left and right hippocampi using z-values calculated using data from either sequence.

An additional analysis within our cohort was performed to determine the number of motion artifacts when comparing the standardized T2 TSE and the accelerated T2 TSE DRB sequences. Two neuroradiologists came to the same conclusion and consistently observed fewer motion artifacts with the accelerated protocol. Overall, the standardized sequence demonstrated four motion artifacts; specifically, one in a patient with hippocampal sclerosis, one in one of two patients with edema, and two among the twenty healthy controls, whereas the accelerated sequence exhibited only a single artifact, observed in one healthy control. Notably, none of the 11 patients with epilepsy without visible pathology, nor the patient with focal cortical dysplasia, showed any motion artifacts on either sequence. These findings suggest that the reduced scan time of the accelerated protocol may significantly diminish motion-related artifacts, thereby potentially enhancing image quality in clinical practice.

## 4. Discussion

Numerous techniques exist to enhance the speed and quality of MRI scans, including improving the signal-to-noise ratio (SNR) and contrast-to-noise ratio (CNR) and minimizing artifacts. These techniques encompass Parallel Imaging, Compressed Sensing, Simultaneous Multislice Imaging, or a combination of these [22,23,24,25]. Emerging methods leveraging artificial intelligence, particularly through convolutional neural networks (CNNs), are being actively tested in clinical settings, showing promising outcomes [5,6,7,26]. Previous studies have predominantly relied on subjective assessments of images reconstructed by DL algorithms using evaluation tools such as Likert scales, along with interrater and intrarater variability analyses to improve objectivity. However, these approaches do not achieve complete objectivity [27,28,29,30].

In contrast, our study exclusively employs numerical volumetric measurements of hippocampal segments using the FreeSurfer program, thereby providing a fully objective assessment of the accelerated imaging technique relative to conventional algorithms. The volumes obtained via the hippocampal segmentation workflow using FreeSurfer exhibited only marginal systematic differences between the accelerated T2 TSE DRB and the standardly used T2 TSE sequence, which did not compromise the detection of pathology on an individual level. These findings objectively support the increased clinical adoption of deep learning algorithms, given their advantages in terms of faster acquisition times and enhanced image quality. Importantly, we focused on accelerating the T2 TSE sequence because it is amenable to the DRB algorithm, unlike the T1 MPRAGE sequence. Although the T1 MPRAGE sequence is critical for delineating gray and white matter and forms an essential part of the neuroimaging protocol recommended by the International League Against Epilepsy, it is currently not available for acceleration using DRB. Conversely, the T2 TSE sequence, also a crucial component of the epilepsy protocol, can benefit from acceleration. By selectively accelerating this sequence, we demonstrated that essential parts of the protocol could be expedited, thereby reducing the overall imaging time while maintaining the requisite diagnostic quality [1].

### 4.1. Comparing T2 TSE DRB with T2 TSE

We demonstrated that a comparison of all hippocampal regions combined and the variance between the T2 TSE DRB and T2 TSE sequences revealed no significant difference, indicating that both sequences exhibit comparable local, spatial, and soft tissue resolution. Therefore, the null hypothesis, stating that there is no difference in the right and left hippocampal volumes when comparing both sequences, is supported by our findings. (Figure 2 and Figure 3).

However, when comparing each subregion and combined label individually, the results were not conclusive. Significant differences in volumes were observed in three segments (CA1-Body, CA4-Body, and whole_hippocampal_body). Upon visual inspection, it was noted that the delineation of these regions varied between the two sequences. This difference could be attributed to the higher signal-to-noise ratio (SNR) and increased image smoothing in DRB-reconstructed images compared to the GRAPPA-reconstructed MRI images used for comparison [13]. Previous studies employing DL sequences in musculoskeletal imaging have suggested that DRB reconstructed images offer sharper boundary delineation and higher diagnostic accuracy than conventional reconstruction methods [5]. This suggests that the T2 TSE DRB sequence may yield more precise images for hippocampal segmentation, which is crucial for the proper functioning of FreeSurfer. Furthermore, it has been demonstrated that the use of new 1.5 mm T2 TSE DRB sequences, as opposed to conventional 1.5 mm T2 TSE sequence images, may enhance lesion detection sensitivity in patients with temporal lobe epilepsy [13].

Noteworthy is that the classification of hippocampal atrophy in hippocampal sclerosis (HS) and alterations in hippocampal regions in patients suffering from epilepsy predominantly occurs in the CA1 and CA4 regions, which exhibit significant differences between both sequences (T2 TSE and T2 TSE DRB). Nevertheless, all pathological volume changes between the left and right hippocampus were reliably identified in our cohort using both sequences. This indicates that the apparent discrepancy between the sequences observed on paper is not clinically relevant. However, our evaluation was limited to a small number of pathologies and subjects, highlighting the need for a standardized approach for a volumetric pathology assessment. Otherwise, false conclusions may be drawn since a valid comparison of the segmented volumes between both sequences may not be possible in these regions. Changing sequences during follow-up evaluations in epilepsy patients is, therefore, not advised [31,32]. We recommend selecting one of the two sequences and conducting all subsequent scans and follow-up evaluations using the chosen sequence.

However, as most segments did not exhibit significant differences when comparing both sequences (T2 TSE and T2 TSE DRB), and the calculated effect size using Cohen’s d was very low (CA1-Body Cohen’s d = 0.0827, CA2/3-Body Cohen’s d = 0.1501), the observed outcomes might be ascribed to random variations. Prior studies have suggested that discrepancies in sequence, hardware, or algorithm may lead to inconsistencies in hippocampal region segmentation conducted by FreeSurfer within the same individual [33]. Additionally, our analysis demonstrated that the accelerated T2 TSE DRB sequence produced four times fewer motion artifacts compared to the standardized T2 TSE sequence, suggesting that the mitigation of motion-related image degradation may even enhance the diagnostic accuracy using the T2 TSE DRB sequence (Table 6).

Utilizing FreeSurfer, hippocampal segmentation can be effectively executed with deep learning (DL)-enhanced sequences of commensurate quality when assessing the overall segmented regional volumes. The adoption of the T2 TSE DRB sequence, which facilitates faster image acquisition, results in heightened patient comfort and image quality and reduced operational costs by increasing the MRI patient throughput. Moreover, this advancement promotes the broader utilization of software applications like FreeSurfer, which not only ascertain the aggregate hippocampal volume but also delineate subregion volumes within it. This methodological approach facilitates a more nuanced evaluation of therapeutic efficacy, age-related alterations, or pathologies impacting the internal hippocampal structure, obviating the necessity for labor-intensive manual segmentation processes.

### 4.2. Testing the 95% CI for Hippocampal Pathology Detection

Using the mean and standard deviation of hippocampal volume difference calculated for every subregion and combined label using healthy individuals, we were able to calculate z-values for hippocampal volume differences within patients, which made it possible to detect pathological volume differences between hippocampi within an individual when z < −2 or z > 2, which corresponds to values lying outside the proposed 95% CI. By exclusively analyzing the within-subject differences, we established a pathology detection range without requiring adjustments for age, sex, and overall brain volume [20]. This streamlined approach accelerated the analysis, allowing for a larger sample size and, therefore, enhancing the precision of the interval. However, this approach presupposes comparable hippocampal volumes between the left and right hippocampi, which were verified prior to establishing the 95% confidence interval.

Notably, our investigation revealed that only a small proportion, specifically 3 out of 44 segments, exhibited significant differences between the left and right hippocampus. This finding is not unexpected, given that healthy individuals can have hippocampal volume discrepancies of up to 10%, whereas patients with temporal lobe epilepsy typically demonstrate an average difference of up to 18% between their hippocampi [19,34,35,36,37]. However, since only a minority of the segments exhibited differences, it can be inferred that the majority of our healthy study population had nearly identical left and right hippocampal volumes, making the creation of an interval for pathological hippocampal volume detection feasible. These results support the null hypothesis, which assumes that there is no significant difference between the volumes of the right and left hippocampus in healthy individuals.

The assessment of subjects with right-sided HS, left-sided FCD with concurrent edema, and one patient suffering solely from right-sided hippocampal edema revealed that the 95% CI (−2 < z < 2) successfully identified pathological regions on the correct hippocampal side, encompassing the majority of combined labels. The inclusion of combined labels in the pathological regions suggests significant pathological variations, as they span the entire hippocampus. By examining regional volumes within the hippocampus, we were additionally able to classify the HS as the primary ILAE Type 1 sclerosis [32]. This illustrates that utilizing the proposed interval and viewing the hippocampal regional volumes can aid in diagnosis and possibly detect overlooked pathology. In that respect, Christian G Bien et. al. demonstrated within his patient population that eight lesions within the hippocampus that had previously been reported as MRI negative had turned out to be visible in MRI after positive post-surgical histological abnormalities had been detected [38]. Our proposed interval may help physicians detect subtle changes that are otherwise overlooked by drawing attention to specific regions through hippocampal volumetry.

Six patients using data from the T2 TSE DRB sequence and seven using data from the T2 TSE sequence, suffering solely from epilepsy with no other comorbidities, revealed z-values outside the 95% CI (−2 < z < 2) when comparing right and left hippocampi. However, none of the combined labels was suspicious in any of these patients, suggesting only subtle hippocampal changes. Subtle alterations undetected previously were visually observed after segmentation but were considered insignificant at the current disease stage. These changes were documented and may offer valuable insights when disease progression occurs. This aligns with prior studies showing that epileptic patients often exhibit localized hippocampal changes not evident across the entire hippocampus [35,39,40,41,42]. Studies have shown that manual regional hippocampal segmentation can help pinpoint the seizure origin in therapy-resistant epilepsy during follow-ups [9]. We further analyzed medical records, including EEG data and clinical diagnoses, in patients exhibiting significant hippocampal asymmetry following z-value analyses. Of the seven patients examined, four had medical records that proposed a hypothesis of seizure origin, while three demonstrated ambiguous seizure lateralization based on EEG and clinical evaluations. In all cases, the seizure origin predicted in the medical records aligned with our findings, with each patient presenting smaller hippocampal volumes on the side corresponding to the proposed seizure origin. Although the underlying etiology remained unclear across all patients, it is widely accepted that various etiologies—such as infarction, infection, or cortical dysplasia—share a common pathological endpoint of sclerosis, consistent with our observations [43].

Unfortunately, one patient suffering solely from FCD was not detected. However, only a minor portion of the hippocampus was involved in the FCD of the temporal lobe, rendering it impractical to draw conclusions about the interval’s efficacy in patients with FCD.

When considering an implementation in a neuroradiologist’s workflow, we would recommend the following diagnostic approach: Firstly, all data should be reviewed by the radiologist. If no pathology is identified on the MRI despite clinical indications, the hippocampal images should be segmented using segmentation software. Subsequently, all segments should be analyzed using the z-score, and regions showing volumetric differences (z < −2 or z > 2) between the right and left hippocampus that lie outside the 95% confidence interval should be re-examined more carefully. We strongly recommend calculating the z-value for each subregion and combined label, as this approach yields more precise assessments (see Table 7). Furthermore, it is important to note that z-values calculated using data from the T2 TSE DRB sequence were able to detect more pathological regions overall within patients, suggesting greater sensitivity, at least in our testing. However, further investigation is warranted to confirm these findings across larger patient cohorts.

### 4.3. Limitations

The limitations of this study include the small number of patients, the retrospective study design, and the limited number and variety of pathological changes involved in comparing the two sequences and testing the 95% confidence interval for functionality. To ensure 100% functionality in all situations, the newly proposed DRB sequence still needs to demonstrate its efficacy using different MRI machines, coil arrangements, and acceleration factors. It is important to note that, with our generated data using healthy controls, z-values can only be derived by comparing the left and right hippocampus in a patient, assuming pathology affects only one side. To calculate the z-values for each hippocampal side independently—facilitating the detection of bilateral pathology—a larger cohort of healthy individuals would be needed. However, most patients present with unilateral mTLE, characterized by distinct features on one side, making our method practical in most cases [44].

## 5. Conclusions

Our study demonstrates that the segmentation of hippocampal subregions and combined labels using the program FreeSurfer can be performed effectively and safely with novel T2 TSE DRB sequences (t2_tse_cor_2mm_DRB). Switching between both sequences (T2 TSE and T2 TSE DRB) during follow-ups is not recommended to improve reliability, especially in epileptogenic patients, despite there being no significant difference overall. In addition, we report the mean and standard deviation of hippocampal volume difference within an individual using healthy controls, which serve as reference metrics for calculating z-scores in patients with suspected pathology. This methodology facilitates the identification of pathological hippocampal volume differences between the right and left hippocampus within an individual by employing a 95% confidence interval (−2 < z < 2), thereby enhancing the detection of significant volumetric abnormalities within a patient. This interval may assist radiologists in the clinical setting by providing a quantitative assessment of hippocampal pathology. Since the interpretation of hippocampal lesions is highly dependent on the expertise and experience of the radiologist, an objective and quantitative assessment may provide a standardized reporting framework for a comparison of the findings. Consequently, the use of the novel T2 TSE DRB sequence enables more rapid image data acquisition, thereby reducing the examination time, enhancing image quality and patient comfort, and diminishing motion artifacts, while simultaneously potentially improving both signal-to-noise and contrast-to-noise ratios [1].

## Figures and Tables

**Table 1:** Presented are detailed patient characteristics, including the average age within each population, the gender distribution within each group, the total number of subjects, and the type of MRI scanner utilized for the scans.

Pathology	Subjects	Gender		Age
		Male	Female	
Hippocampal sclerosis	1 (3%)	0 (0%)	1 (100%)	57 ± 0
FCD ^a^	2 (6%)	2 (100%)	0 (0%)	42 ± 28
Edema ^b^	2 (6%)	2 (100%)	0 (0%)	29 ± 9
Epilepsy (without visible pathology)	11 (31%)	6 (55%)	5 (45%)	41 ± 13
Healthy	20 (56%)	11 (55%)	9 (45%)	37 ± 15
Overall	36 (100%)	21 (58%)	15 (42%)	39 ± 14

^a^ Focal cortical dysplasia; ^b^ T2 hyperintensity with edema. All patients were scanned using the MRI MAGNETOM Vida Fit 3T from Siemens Healthcare (Erlangen, Germany).

**Table 2:** This table contains the MRI settings specified for each sequence.

		Sequence		
		T1 3D MPRAGE	T2 2D TSE	T2 2D TSE DRB
MRT Settings	Slice orientation	sagittal	coronal	coronal
	Slices	192	35	35
	Acceleration	GRAPPA ^d^ R = 2	GRAPPA ^d^ R = 2	GRAPPA ^d^ R = 4
	Reconstruction	GRAPPA ^d^	GRAPPA ^d^	DRB ^e^
	Slice thickness (mm)	0.90	2.00	2.00
	TR ^a^ (ms)	2300	4100.00	4100.00
	TE ^b^ (ms)	2.32	76.00	76.00
	Flip Angle (deg.)	8	150.00	150.00
	Deep Resolve	OFF	OFF	ON
	Concatenations	1	2	2
	Voxel size (mm)	0.9 × 0.9 × 0.9	0.2 × 0.2 × 2	0.2 × 0.2 × 2
	Averages	1	3	3
	Distance factor (%)	50	0	0
	FOV ^c^ Read (mm)	230	170	170
	FOV ^c^ Phase (%)	100	100	100
	Phase Resolution (%)	100	85	85
	Trajectory	Cartesian	Cartesian	Cartesian
	Bandwidth (Hz/Px)	200	200	200
	Echo Spacing (ms)	7.06	10.9	10.9
	Dimensions	3D	2D	2D
	RF Pulse Type	Normal	Normal	Normal
	Gradient Mode	Normal	Fast	Fast
	Flow Compensation	None	Read	Read
	Turbo Factor	240	19	19
	Phase Oversampling (%)	0	0	0
	Slice Oversampling (%)	25	/	/
	Base Resolution	256	384	384
	Slice Resolution (%)	100	/	/
	Reference Lines	24	44	44
	Coil Selection	automatic	automatic	automatic
	Coil Combination	adaptive	adaptive	adaptive
	Echo Trains per Slice	/	6	6
	Time (min.)	5:21	3:51	2:37

^a^ Repetition Time; ^b^ Echo Time; ^c^ Field of View; ^d^ Generalized Autocalibrating Partial Parallel Acquisition; ^e^ Deep Resolve Boost.

**Table 3:** This table compares the incidence of motion artifacts, evaluated by two board-certified neuroradiologists, between two MRI sequences, DL accelerated (T2 TSE DRB) and standardized (T2 TSE), across different patient groups. For example, the single patient with hippocampal sclerosis exhibited a motion artifact only in the T2 TSE sequence, while neither of the two patients with focal cortical dysplasia showed any artifacts in either sequence. In the edema group, one of two patients had a motion artifact in the T2 TSE sequence. Patients with epilepsy without visible pathology had no artifacts in either sequence. Among the healthy controls, one artifact was observed with the T2 TSE DRB sequence versus two with the T2 TSE sequence. Overall, the total count reveals that the T2 TSE DRB sequence produced fewer motion artifacts (1 vs. 4), underscoring its potential for improving image quality and diagnostic accuracy by reducing the impact of motion artifacts.

Pathology	*n*	Accelerated (T2 TSE DRB *)—Motion Artifacts	Standardized (T2 TSE ^+^)—Motion Artifacts
Hippocampal Sclerosis	1	0	1
Focal Cortical Dysplasia (FCD)	2	0	0
Edema	2	0	1
Epilepsy (without visible pathology)	11	0	0
Healthy Controls	20	1	2
Total	36	1	4

* Deep Resolve Boost; ^+^ Turbo spin echo.

**Table 4:** This table presents the mean and standard deviation (SD) of volume differences between the right and left hippocampus for each subregion and combined label in healthy individuals. These reference values can be used to calculate z-scores, aiding in the detection of pathological volume differences between the right and left hippocampus in patients. Z-scores below −2 or above 2, which correspond to values outside of a 95% confidence interval, indicate significant volumetric differences between the right and left sides of a specific hippocampal region, suggesting potential pathology. Use the following formula to calculate z-scores for specific subregions or combined labels in patients (values for Mean_healthy_ and Standard deviation_healthy_ should be taken from this table). Note that cells highlighted in red belong to subregions (first 19 cells), whereas cells highlighted in blue (last 3 cells) belong to combined labels. $z=\frac{\left(right\;hippocampal\;region_{patient}-left\;hippompal\;region_{patient}\;\right)-Mean_{healthy}}{Standard\;deviation_{healthy}}$.

Region	T2 TSE ^‡^ DRB ^†^ Sequence		T2 TSE ^‡^ Sequence	
	MEAN_healthy_ (mm^3^)	SD_healthy_ * (mm^3^)	MEAN_healthy_ (mm^3^)	SD_healthy_ * (mm^3^)
Parasubiculum	−7.6	13.8	−7.2	13.8
Presubiculum-Head	−6.3	16.3	−5.9	13.1
Subiculum-Head	−2.5	18.9	−2.6	18.2
CA1-Head	11.0	36.8	9.3	40.5
CA2/3-Head	9.4	17.7	7.8	17.3
CA4-Head	5.2	14.0	7.4	13.4
GC-ML-DG-head	6.3	18.6	9.0	18.6
molecular_layer_HP-head	−2.1	25.0	−4.7	23.8
HATA	0.7	8.1	0.5	8.8
Presubiculum-body	−19.0	22.5	−18.8	20.0
Subiculum-body	−13.5	18.0	−15.6	14.2
CA1-Body	9.1	23.2	4.5	22.6
CA2/3-body	7.4	16.5	5.5	17.7
CA4-body	−0.8	11.8	−0.2	12.3
GC-ML-DG-body	−1.1	14.9	−0.7	16.0
molecular_layer_HP-body	7.4	25.0	11.3	23.9
fimbria	0.1	17.0	0.1	19.5
Hippocampal_tail	0.7	57.0	0.9	56.0
hippocampal-fissure	−3.0	18.5	−3.6	19.9
Whole_hippocampal_body	−10.6	74.5	−13.8	70.6
Whole_hippocampal_head	13.9	107.0	13.5	99.5
Whole_hippocampus	4.0	194.9	0.6	181.9

* Standard deviation; ^†^ Deep Resolve Boost; ^‡^ Turbo spin echo.

**Table 5:** One can observe the *p*-values for volumetric comparisons of segments within the hippocampus between T2 TSE and T2 TSE DRB sequences. Furthermore, the mean volume of each region and the standard deviation of volume are provided in mm^3^. The *p*-values for comparing the left and right hippocampus are also provided and can be located in the right section of the table. Note that cells highlighted in red belong to subregions (first 19 cells), whereas cells highlighted in blue belong to combined labels (last 3 cells).

Region	T2 TSE ^a^ vs. T2 TSE DRB ^b^									
	Right Hippocampus					Left Hippocampus				
	Mean Volume T2 TSE (mm^3^)	Mean Volume T2 TSE DRB (mm^3^)	Mean Volume Difference (mm^3^)	SD ^c^ (mm^3^)	*p*-Value	Mean Volume T2 TSE (mm^3^)	Mean Volume T2 TSE DRB (mm^3^)	Mean Volume Difference (mm^3^)	SD ^c^ (mm^3^)	*p*-Value
Parasubiculum	63.9	64	0.2	1.6	1	66.9	65.9	−1	2.1	0.726
Presubiculum-Head	124.4	123.5	−0.9	4	1	128.6	129.5	0.9	5.9	1
Subiculum-Head	186.4	185.8	−0.6	4.2	1	188.4	188.3	−0.04	4.2	0.958
CA1-Head	566	569.9	3.9	9.6	0.55	556.8	559.2	2.4	9.6	1
CA2/3-Head	137.3	136.9	−0.4	3.7	1	130	128.7	−1.3	3.5	0.871
CA4-Head	141.2	139.7	−1.5	2.9	0.158	134.9	134.6	−0.2	3.9	1
GC-ML-DG-head	174.2	172.5	−1.7	3.5	0.234	166.8	166.7	−0.1	4.7	1
molecular_layer_HP-head	323.5	324.6	1.1	10.3	1	341	337.6	−3.4	11.3	1
HATA	66.1	65.4	−0.6	1.9	1	67.4	64.6	−2.8	12.8	1
Presubiculum-body	139.3	140.2	0.9	3.9	1	157.2	158.8	1.5	4.7	1
Subiculum-body	232.9	232.7	−0.3	4.3	1	250.1	249.3	−0.8	6	1
CA1-Body	**133.2**	**135.2**	**2**	**2.6**	**0.003**	130.1	128.9	−1.1	4.3	1
CA2/3-body	92.4	91.5	−0.9	4	1	89.2	87	−2.2	4.1	0.126
CA4-body	**116.1**	**113.1**	**−3**	**3.3**	**0.0002**	116.3	114.8	−1.5	3.5	0.55
GC-ML-DG-body	129.6	130.5	0.9	20.5	1	130.8	130.2	−0.6	4.5	1
molecular_layer_HP-body	250	248.2	−1.8	23.3	1	239.9	238.2	−1.8	6.9	1
fimbria	73	74.3	1.4	3.4	0.681	72.6	74.9	2.3	4.1	0.079
Hippocampal_tail	564.5	564.7	0.2	6.4	1	561.7	563.1	1.3	6.4	1
hippocampal-fissure	138.8	138.5	−0.4	4.3	1	135.6	137.5	1.9	17.1	1
Whole_hippocampal_body	**1166.5**	1158.7	**−7.8**	**13.8**	**0.012**	1186.2	1182	−4.2	15.7	0.491
Whole_hippocampal_head	1782.9	1782.5	−0.4	19.1	0.897	1778.6	1777.1	−1.5	19.9	1
Whole_hippocampus	3513.9	3505.9	−8	27.8	0.487	3526.5	3522.1	−4.3	33.6	1

^a^ Turbo spin echo; ^b^ Deep Resolve Boost, ^c^ Standard deviation.

**Table 6:** One can observe the *p*-values that were calculated when comparing the volumes of subregions and combined labels between the right and left hippocampus, once using the T2 TSE sequence and once using the T2 TSE DRB sequence. Note that cells highlighted in red belong to subregions (first 19 cells), whereas cells highlighted in blue belong to combined labels (last 3 cells).

Region	Right Hippocampus vs. Left Hippocampus	
	T2 TSE ^a^	T2 TSE ^a^ DRB ^c^
	*p*-Value	
Parasubiculum	0.927	0.774
Presubiculum-Head	1	1
Subiculum-Head	1	1
CA1-Head	1	1
CA2/3-Head	1	0.929
CA4-Head	0.76	1
GC-ML-DG-head	1	1
molecular_layer_HP-head	1	1
HATA	1	1
Presubiculum-body	**0.018**	**0.045**
Subiculum-body	**0.004**	0.117
CA1-Body	1	1
CA2/3-body	1	1
CA4-body	1	1
GC-ML-DG-body	1	1
molecular_layer_HP-body	1	1
fimbria	1	1
Hippocampal_tail	1	1
hippocampal-fissure	1	1
Whole_hippocampal_body	1	1
Whole_hippocampal_head	1	1
Whole_hippocampus	0.988	1

^a^ Turbo spin echo; ^c^ Deep Resolve Boost.

**Table 7:** This table demonstrates how z-values can be calculated for a patient suffering from right-sided hippocampal sclerosis. By using the given formula for z-value calculation and entering the reference values from Table 6 along with the volume difference between the right and left hippocampus for the patient of interest, z-values can be easily calculated. Volume differences with z-values exceeding z > 2 or −2 < z are defined as pathological—marked as bold in the table. As demonstrated in this example, all combined labels as well as most subregions were identified as pathological on the correct side in this patient with right-sided hippocampal sclerosis. Note that cells highlighted in red belong to subregions (first 19 cells), whereas cells highlighted in blue belong to combined labels (last 3 cells).

Region	Patient 01—Hippocampal Sclerosis Right			
	Right Hippocampus (mm^3^)	Left Hippocampus (mm^3^)	RH *-LH ^†^ (mm^3^)	z-Value
Parasubiculum	33.0	53.7	−20.7	−0.9
Presubiculum-Head	70.6	108.1	−37.6	−1.9
Subiculum-Head	101.7	148.1	−46.4	**−2.3**
CA1-Head	251.1	437.4	−186.3	**−5.4**
CA2/3-Head	62.8	99.8	−37.0	**−2.6**
CA4-Head	53.4	99.9	−46.5	**−3.7**
GC-ML-DG-head	67.3	123.1	−55.8	**−3.3**
molecular_layer_HP-head	151.9	251.8	−99.9	**−3.9**
HATA	44.7	53.3	−8.6	−1.1
Presubiculum-body	73.2	132.1	−58.9	−1.8
Subiculum-body	121.9	216.7	−94.8	**−4.5**
CA1-Body	67.2	130.3	−63.1	**−3.1**
CA2/3-body	39.2	82.3	−43.1	**−3.1**
CA4-body	40.6	103.4	−62.7	**−5.2**
GC-ML-DG-body	46.7	117.4	−70.7	**−4.7**
molecular_layer_HP-body	145.8	239.4	−93.7	**−4.0**
fimbria	24.4	32.9	−8.5	−0.5
Hippocampal_tail	288.5	486.2	−197.6	**−3.5**
hippocampal-fissure	101.1	126.3	−25.2	−1.2
Whole_hippocampal_body	559.2	1054.5	−495.3	**−6.5**
Whole_hippocampal_head	836.5	1375.2	−538.8	**−5.2**
Whole_hippocampus	1684.2	2915.9	−1231.8	**−6.3**

* Right hippocampus; ^†^ Left hippocampus.

## Data Availability

The data that support the findings of this study are not openly available due to reasons of sensitivity and are available from the corresponding author upon reasonable request.
